# Micronutrients Deficiency, a Hidden Hunger in Nepal: Prevalence, Causes, Consequences, and Solutions

**DOI:** 10.1155/2015/276469

**Published:** 2015-01-15

**Authors:** Shiva Bhandari, Megha Raj Banjara

**Affiliations:** ^1^Multivitamin-Mineral Supplementation Project, Health Resources Consultancy Pvt. Ltd., Kuleshwor, Kathmandu 44614, Nepal; ^2^Central Department of Microbiology, Tribhuvan University, Kirtipur, Kathmandu 44618, Nepal

## Abstract

Micronutrient deficiency is a global challenge to health as in Nepal. In Nepal, the targeted beneficiaries are less aware about importance of micronutrients (MNs), which has resulted in low intake of foods rich in MNs. Micronutrient deficiencies (MNDs) have huge impact on health of vulnerable population like women and children and have jeopardized the national economy and prosperity of developing countries including Nepal. However, less attention has been paid towards MNDs, which can be prevented. Therefore, this study aims to draw attention of the concerned authorities and researchers to combat against MNDs in Nepal. This study showed that different types of MNDs with higher prevalence exist in Nepal. The major causes of MNDs were poor diet, diseases and infestations, and poor health caring practices. The results of MNDs were unwanted child and maternal mortality, impairments of lives, and reduction in productivity and intellectual capacity. School health and nutrition education and supplementation and fortification of essential MNs proved to be effective while dietary diversification and economic growth and poverty eradication seemed promising. Control and prevention of MNDs can help to achieve Millennium Development Goals as well, so studies in this sector should be emphasized.

## 1. Introduction

Apart from the protein-energy malnutrition (PEM, which includes marasmus and kwashiorkor), there exists another form, which is less visible and a result of vitamins and minerals deficiencies, known as micronutrient deficiency (MND) [1]. If people do not get sufficient food to eat, they are malnourished let alone getting MNs. Therefore, MND can be regarded as a subset of malnutrition. Deficiencies of fat soluble vitamins, iron, and zinc are particularly common, but deficiencies of other water-soluble vitamins, minerals, and trace elements are also found and have great impact in physical, mental, and cognitive development of an individual. Iron deficiency is the most prevalent nutrition problem in the world [2]. Folic acid deficiency remains responsible for excess birth defects [3]. Vitamin D deficiency can lead to osteoporosis and bone fractures and may become life-threatening or leave an elderly person permanently handicapped, thus reducing length and quality of life [4]. Vitamin A deficiency is a public health problem in more than half of all countries, especially in Africa and Southeast Asia, which causes preventable blindness and increases the risk of disease and mortality [5].

Micronutrients (MNs) (vitamins and minerals) are essential for proper growth and development apart from macronutrients (carbohydrates, fats, and proteins). MND has global health impact because its manifestations become less visible and usually begins to show when the condition is severe and has already led to serious health burdens, justifying the name “hidden hunger.” Deficiencies occur when people do not have access to micronutrient-rich foods such as fruit, vegetables, animal products, and fortified foods, usually because they are too expensive to buy or are locally unavailable. Although the deficiency affects every age group of both sexes, the most vulnerable groups are children and women of reproductive age including pregnant and lactating mothers [6]. The World Health Organization (WHO) estimates that more than 2 billion people are deficient in key vitamins and minerals, including a third of world's children [7]. Most of these people live in low-income countries and the situation can be even worse in Nepal. However, its magnitude is not clear due to insufficient research data from the nutritional surveys.

## 2. Prevalence

Although there are several MNDs prevailing in Nepal, only very few deficiencies have been studied, which has been focused on only in the vulnerable groups. National data regarding all MNDs is still lacking. It is very necessary to measure the prevalence of MNDs as they can have serious effects on health, education, and economic prosperity. In addition, the lack of one MN can result in the deficiency of another MN [8].  Table 1 summarizes the overall prevalence of some MNDs and the deficiency disorders.

## 3. Causes

**Figure 1:**
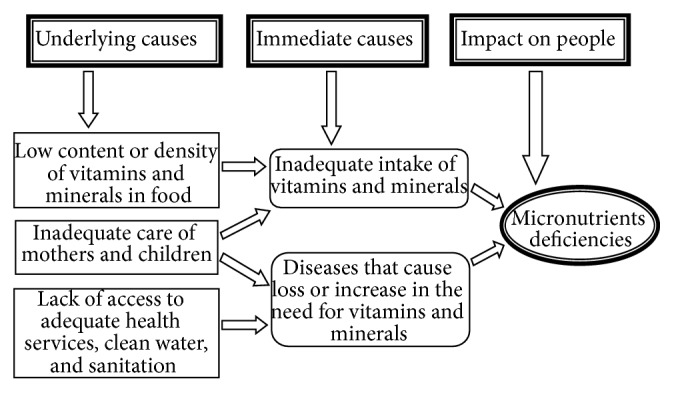
Interlinkage among the causes of MNDs in Nepal.

The causes of micronutrients deficiencies are multiple and interconnected (Figure 1).

### 3.1. Improper Diet

The most immediate cause of MNDs is poor nutrient intake through inadequate diets [9]. The principal Nepalese diet consists of rice and bread, which can provide carbohydrate but not MNs, which occur naturally in foods like meat, eggs, fish, milk, legumes, fruits, and vegetables. However, not all people in Nepal are privileged to have such foods and those who have access do not consume them regularly. Micronutrient deficiencies likely coexist where diet is poor because of expense or limited or seasonal availability of food [10]. Despite its fundamental importance, improving the diets of the people of resource poor nations is a complex and long-term undertaking that depends largely on rising incomes.

### 3.2. Diseases and Infestations

The body's ability to absorb and retain MNs decreases with diseases [11]. It can even lead to actual losses of them, as in the case of zinc and other minerals loss during diarrhea. Vitamin and mineral nutrition is severely compromised by parasitic infestations such as hookworm [12]. The deficiencies caused by diseases leave the individuals more vulnerable to further illness and less able to absorb MNs.

### 3.3. Underlying Causes

The underlying causes of MNDs are insufficient access to food, inadequate health care, and poor caring practices that inhibit growth and health [9]. In Nepal, more than a quarter of the population consumes inadequate food and the average household food can meet their food needs for only about four months [13]. Similarly, the ineffective health care system and poor caring practices still prevail in most of the rural parts of Nepal. The content of the micronutrients in the food always raises the question of MNDs. The provision of nutrition and child-care education, particularly to women, is also essential. Despite the many causes of MNDs and the great challenge posed by the sheer numbers of people affected by them, the government is languid to address the problems.

## 4. Consequences

**Figure 2:**
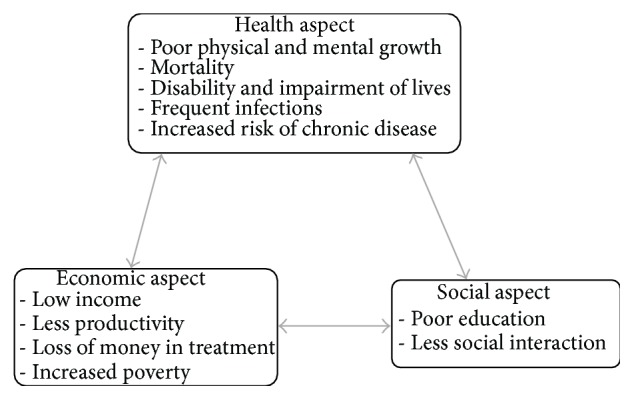
The vicious cycle of MNDs.

The MNDs always have negative effects in physical and mental health [14, 15], which hinders the social progress and economical prosperity of an individual and nation as a whole. This condition perpetuates the vicious cycle of poor economic growth, health impairments, and low social status (Figure 2).

### 4.1. Child and Maternal Mortality

Unnecessary child and maternal deaths are the most unacceptable effects of MNDs in developing nations like Nepal. Mostly death comes with pregnancy and birth, and it comes more after battles with disease. According to an analysis of the 2011 survey in Nepal, the odds of neonatal death are higher for babies whose mother is of short stature, compared with babies whose mother is of normal stature, and the neonatal mortality rate in 2011 was 31 deaths per 1,000 live births due to anemia in mothers [14]. Iron deficiency anemia (IDA) during pregnancy is associated with 115,000 women's deaths each year [15], which account for one-fifth of total maternal deaths [16]. Each year vitamin A deficiency (VAD) claims the lives of almost 670,000 children under five in the world [15] and precipitates the deaths of approximately 6,900 children in Nepal [17]. Annually, zinc deficiency claims more than 450,000 lives of children [15] and poses threat to the lives of children and women in Nepal. In addition, zinc deficiency in mothers is detrimental to both mothers and child [18], especially during prenatal and early postnatal development [19]. Most babies with anencephaly, a serious neural tube defect, due to folic acid deficiency before conception do not survive birth.

### 4.2. Impairment of Lives and Disability

Although the number of children and women who die because of MNDs is great, the number of people who live with these deficiencies and their consequences is still greater [9]. They suffer from not only multiple MNDs but also multiple impairments. In Nepal, disability can be a devastating burden for individuals and their families who lack resources and the options for learning and income earning are limited. There is always higher risk of death due to infections in night-blind pregnant women than those who are not [20]. Each year in Nepal, VAD is responsible for the deaths of 9000 children and for 2500 children becoming permanently blind [21]. These children face daunting physical, social, and ultimately economic challenges. Each year, neural tube defects (NTDs) affect about 300,000 newborns worldwide, half of which can be prevented by folic acid intake before pregnancy [22]. According to recent unpublished research data, the prevalence of NTDs in Nepal is 1.1 per 1000 live births. Rigorous research is required to assess the prevalence, consequences, and strategies to prevent such defects in Nepal.

### 4.3. Reduction in Intellectual Capacity

Reduced intellectual capacity undermines investments in education and perpetuates cycles of poverty. It is a significant barrier for any nation to achieve economic growth and improved standards of living. Iodine deficiency in pregnancy is the greatest cause of preventable mental impairment in the world [23]. In Nepal, iodine deficiency in pregnancy causes more than 200,000 babies a year to be born mentally impaired; even mildly or moderately iodine-deficient children have IQs that are 10 to 15 points lower than those who are not deficient [17]. Intellectual ability is also affected by deficiency of iron and zinc [24, 25].

### 4.4. Loss of Productivity

There is unwanted loss of productivity in national economy every day due to MNDs. The economic growth is staggering in countries having the highest numbers of people living with physical and intellectual impairments [26]. About 2-3% of GDP is lost every year in Nepal on account of vitamin and mineral deficiencies alone [17]. The laborers who become sick or have disabled children lose days of work. Adults living with reduced energy and intelligence are unable to fully contribute to society.

### 4.5. Burden on Caregivers and Health Systems

There is unnecessary burden on professional caregivers to serve for those who have been suffering from one or multiple preventable MNDs [9]. The time and resources of health care providers spent in the diagnosis and treatment of children because of MNDs can make the health system work slow. Childhood illness, especially when frequent and long, can lead to unaffordable costs for many families, in terms of both drug treatment and productive time lost in caring for the ills.

## 5. Prevention and Control of MNDs

The control of MNDs should no longer be focused on one deficiency at a time but multiple MNDs simultaneously, which can be operationally effective.

### 5.1. School Health and Nutrition (SHN) Education

School is the first place where one can learn about advantages of better nutrition. The major advantage of school education on nutrition is behavior change and its sustainability [27]. When the target audience is motivated and educational intervention is well designed and delivered, the chances of success are high. In Nepal, SHN strategy came into action in Syangja and Sindhupalchowk in 2006 with expansion to other districts afterwards. Currently there are several external development partners (EDPs) like JICA, Save the Children, CCS Italy, UNICEF Nepal, Plan Nepal, and Helen Keller International and NGOs like Nepal Red Cross Society and Nepal Water for Health (NEWAH) working in their priority areas of SHN in some VDCs of the selected districts. The Ministry of Health and Population has initiated school deworming program in 45 districts including two pilot districts in 2009/10 and prepared guidelines for celebrating a SHN Week all over the country since December 2009. Likewise, the Ministry of Education has initiated students feeding programs in 30 districts (as cash model in 19 districts and kind model in 11 districts). Each year around 5000 school toilets with water supply are constructed in 75 districts of Nepal. After the implementation of SHN related programs, rate of worm has been declined from 27% to 7%, and hygiene behaviors of the students have been improved [28]. All these are the outcomes of SHN program in the country and such activities should be scaled up. Despite the involvement of the various organizations including the government, the present SHN initiatives are weak and inadequate to address the health and nutrition needs of school students at the national level. Besides, school education strategies should be worked out to provide nutrition education to illiterate women and head of households in the communities.

### 5.2. Dietary Diversification

In the nations like Nepal where majority of people depend upon dal-bhat (pulse-rice), and the food prices are soaring daily, it becomes difficult for an average Nepalese to have normal balanced diet and there are high chances of suffering from MNDs. A recent annual health report by the government of Nepal [29] presented limited information regarding dietary diversification in Nepal. However, there are several instances where dietary diversification can help to reduce MNDs [30–32]. The strategies employing agricultural interventions, animal husbandry, or aquaculture have the potential to increase intakes of total or absorbable zinc [33]. Similarly, a systematic review [34] showed that with the increased dietary diversity there is increased consumption of *β*-carotene rich vegetables and fruit, increased intake of other vegetables and fruit, increased intake of legumes, and improved complementary foods. Diversification of crop cultivation and making a wider selection of foods with a high vitamin and mineral content available for purchase can be adopted so that the consumers prepare more varied meals and have a more balanced diet [35].

### 5.3. Supplementation

The government of Nepal supplements very few essential MNs like vitamin A, iron/folic acid, and zinc for certain duration only to vulnerable populations [36]. Supplementation of vitamin A for children under five years of age under vitamin A supplementation (VAS) program twice a year by female community health volunteers (FCHVs) through a “campaign-style” activity has proved to be successful [37] and cost effective. Supplementation of iron/folate at no cost to pregnant women and lactating mothers through the network of government health system has uneven results due to limited coverage, shortage of the iron/folate tablets at the community level, and low compliance [29]. Since 2005, the government has been providing zinc to manage diarrhea among children, which, however, needs monitoring and evaluation at regular intervals. Supplementation depends upon a viable delivery system with built-in quality control, as well as wide coverage and high acceptance rates among vulnerable individuals and families [9]. Moreover, supplemental administration of zinc can expedite the healing process and results in faster resolution of clinical symptoms in children with pneumonia [38]. In Iran, the government has been supplementing iron to school girls and has started supplying vitamin D to them [39]. Similarly, zinc syrup (containing 5 mg elemental zinc) supplemented to children less than two years from primary health center in Iran showed effective increase in linear growth [40]. Supplementation only works if the supplements are available and accessible and the intended individuals actually take them. Despite the fact that supplementation of vitamin A, zinc, and iron/folate has positive impact on health of the vulnerable population, there are other various MNs that are equally essential and should be incorporated into national supplementation program. Therefore, the government and concerned organizations need to be serious using multiple MN supplementations, along with the traditional iron/folate and vitamin A, and monitor the compliance rate.

### 5.4. Fortification

Food fortification with single, dual, or multiple micronutrients is a public health approach that has been widely used and is potentially an effective strategy to address micronutrient malnutrition [41]. In addition, food fortification is very economical that each dollar invested in salt iodization returns US$30 in benefits [9]. In the USA annual fortification costs approximately US$3 million and direct medical costs averted are $145 million per annum; consequently, $48 are saved annually for every dollar spent on fortification [42]. Therefore, food fortification can be very cost effective even in resource poor nations like Nepal. For better outcome, fortification of foods can be conceptualized as central or peripheral. Central fortification of foods is done with MNs added in commercial or other central processing, prior to distribution or marketing, while peripheral fortification involves the addition of MNs to foods at household or other consumption level. In Nepal, the latter approach is cost effective and has been adopted by the government and other organizations like Suaahara. Although the former approach is promising, it is a huge success as in case of reduction of iodine deficiency by fortification of salt with iodine [36]. There are also other foods such as biscuits, noodles, cookies, oil, sugar, flour, milk, and tea that are consumed by almost all the population and can be used as fortification tools; however universal consumption should be guaranteed. In a randomized trial an addition of iron- and riboflavin-rich powder to school meals in India reduced anemia in five- to nine-year-old children by more than fifty percent, as well as essentially eliminating clinical signs of riboflavin deficiency [43]. The major advantage of food fortification is that no or minimal behavior change is required on the part of the population provided that safe and effective levels of the relevant nutrients can be delivered through an appropriate food vehicle. This provides a tremendous advantage in terms of coverage and efficiency. However, fortification of foods requires active governmental leadership, policy, and political will, which is currently lacking in Nepal.

### 5.5. Economic Growth and Poverty Eradication

Whatever other causes may be, the root cause of MNs malnutrition is poverty and sluggish economic growth and perpetuating the vicious cycle of poverty and malnutrition [44]. General economic growth is a lagging predictor of nutritional improvement. It is important to break the vicious cycle of poverty and MNs malnutrition in order to prosper. A recent report from Food and Agriculture Organization (FAO) [45] summarizes that policies aimed at enhancing agricultural productivity and increasing food availability, especially when smallholders are targeted, can achieve hunger reduction even where poverty is widespread, which can guide policy makers in Nepal. Analysis of large data set based on Demographic and Health Surveys in a number of countries has summarized that anemia rates do decrease as incomes increase [46]. At present, some programs like Knowledge-based Integrated Sustainable Agriculture and Nutrition (KISAN) and Agriculture and Food Security Project (AFSP) are working parallel with nutrition and income generating activities. The government should strengthen and promote such programs and invite similar other programs to come into action.

### 5.6. Integrated Approaches

Integrated programs seem to be more successful to address the problems of MNDs in developing nations like Nepal [47]. Mother and Child Health Care Program focusing on infants, children, and women; Integrated Management of Acute Malnutrition (IMAM) Program for preventing mortality and morbidity due to malnutrition; and supportive programs like Suaahara, KISAN, and AFSP are working to combat against MNDs along with income generation, agriculture, and sanitation and hygiene. Although rigorous research regarding the benefits of integrated effort of such programs has not been done, the government should promote them and strengthen their activities.

## 6. Discussion

The overall prevalence of MNDs in Nepal ranges from 6.2% to 59.0% with different rates for different types of micronutrients. Similar scenario can be seen from the studies in developing countries [53] like India [54]. However, in developed countries the prevalence of MNDs is very low and is limited to iron deficiency [55, 56]. Iron deficiency anemia among children in rural Africa is most often believed to be the result of nutritional deficiencies [57]. Folate deficiency has been described in South Africa [58, 59]. Although further clarification is necessary, vitamin A deficiency may contribute to anemia in pregnancy [60, 61]. Vitamin B12 deficiency was found in 88% of the cases in a study done in Uttarakhand, India [62]. In Nepal, due to limited research, prevalence of some MNs could not be assessed. As the Government of Nepal is focusing on some fat soluble vitamins (vitamin A and vitamin D) and minerals (iron, iodine, and folic acid), it is always prudent not to underestimate the benefits of other MNs as well.

The overall cause of MN malnutrition is lack of proper diet. In general places of Africa and South Asia, many children suffer from malnutrition primarily due to shortage of food [63, 64]. The condition is aggravated by certain diseases and parasitic infestations, which reduce the capacity of body to retain MNs [11, 12]. In a study done in India, worm infestation was recorded in the children suffering from PEM [65]. Besides, ineffective health care system, poor MN content in food, poor caring practices, and unsatisfactory nutrition education play a role in MN malnutrition. For example, a study conducted in West Bengal, India, suggested that provision of nutrition education to mothers of infants had a positive effect on the nutritional status of their children [66]. Therefore, nutrition education should also be prioritized.

There have been always grave consequences of MNDs especially among children and pregnant mothers. There is increased changes of maternal and child morbidity and mortality with the increase in MNDs in Nepal [14–17]. In a study, Rush reviewed there was increased maternal mortality due to undernutrition in developing countries [67]. When people suffer from multiple MNDs, they are at higher risk for multiple impairments. A study showed that iodine, iron, and zinc deficiencies are associated with cognitive deficits among children [68], primarily due to iodine deficiency [23]. One should not forget the negative impacts of physical and mental impairment in national economy [26]. Results from case study in Sierra Leone estimated that anemia among women was associated with agricultural productivity losses of US$19 million per year [69]. Similarly, children who are malnourished are more likely to start school late, to perform less well, and to stay in school for a shorter time [70].

As education is a way to change one's behavior, school health and nutrition education has always been promising endeavor in controlling MNDs in Nepal. Nutrition education has been able to reduce malnutrition among children in Pakistan [71]. In a study done in Tanzania and Zimbabwe, child wasting was higher in the mothers having lower level of education [72]. Similarly, studies showed that child stunting was highest for children whose mothers had no schooling and was lowest among children whose mothers had higher education level in Kenya [73], Zimbabwe [74], and Malawi [72]. These results imply that education is one of the important factors in reducing malnutrition and thus MNDs. There have been motivating results in intake of MNs due to dietary diversification program in Bangladesh, Cambodia, Nepal, and Philippines, which integrates home gardening with animal husbandry, has led to a substantial increase in consumption of animal food along with plant food, and has reduced the prevalence of childhood anemia [32]. It is always judicious to adopt interventions that can promote such diversification. There are various forms of diversification like promotion of mixed cropping and integrated farming systems; promotion of improved preservation and storage of fruits and vegetables to reduce waste; postharvest losses and effects of seasonality; promotion of fishery and forestry products for household consumption; promotion of underexploited traditional foods and home gardens; small livestock raising; strengthening of small-scale agroprocessing and food industries; and introduction of new crops (such as golden rice). When the dietary diversification is community-based, culturally acceptable, and economically feasible, it has the potential to be the most sustainable long-term intervention of all for preventing multiple MNDs within an entire household and across generations.

Although supplementation of very essential MNs to vulnerable population [36] improves their health, other groups of population should not be ignored in formulating policies in Nepal. Due to poor results from national micronutrient survey, 2001 [75], Iranian Ministry of Health, with the cooperation of the Ministry of Education, implemented an Integrated Iron Deficiency Control Program (IDCP), targeting high school girl students. In a study of children aged 7 to 10.5 years in an Indian school, supplementation of 19 key vitamins and minerals in fortified choco-malt beverage improved micronutrient status and enhanced aerobic capacity and endurance in the children [76]. WHO has also recommended the intermittent iron and folic acid supplementation in menstruating women living in settings where anemia is highly prevalent, to improve their hemoglobin concentrations and iron status and reduce the risk of anemia [77]. Another cost effective strategy is food fortification, which has been adopted by Nepal and similar other resource limited nations. Salt fortification with iodine has been successful in Nepal [36]. Fortified white sugar has been successful in reducing VAD prevalence in Central America [78]. Similarly, there was a significant improvement in folate status in women of childbearing age approximately nine months after fortification of maize and wheat foodstuffs in South Africa [59]. Apart from these, poverty eradication is another factor in reduction of MNDs. Although income growth does improve nutrition, macroeconomic policies alone will not suffice to reduce hunger and achieve other MDGs, and nutrition interventions are necessary to address nonincome poverty [79]. Despite the fact that integrated programs prove to be more successful in reducing MNDs [47], more studies have to be done.

## 7. Conclusions

At present the Government of Nepal along with other organizations is working to combat against MNDs in Nepal. However, the effects in the national level are still lacking. Therefore, it is the responsibility of the government to scale up and promote effective programs. In addition, there is a need of an effective body for surveillance, research, and monitoring of MNDs in the nation. Without these steps, we are going nowhere. While it will be imperative to scale up direct nutrition interventions, success will be enhanced and sustained by addressing underlying determinants of nutrition through action in multiple sectors such as poverty alleviation, education, agriculture, social protection, water, and sanitation. Furthermore, an improved mechanism is required within the National Planning Commission to coordinate the varied multisectoral activities to improve nutrition.

## Figures and Tables

**Table 1:** Micronutrients deficiency prevalence and the major disorders.

Micronutrients	Deficiency prevalence	Major deficiency disorders
Iron	35% of women (15–49 years of age) and 46% of children (under five years) [36]	Iron deficiency anemia, reduced learning and work capacity, increased maternal and infant mortality, low birth weight, impaired human function at all stages of life
Iodine	22.0–27.9% [48–50] (urinary iodine <100 *μ*g/L)	Cretinism, goiter, impaired cognitive function, increased prenatal morbidity and mortality, reduced productivity
Zinc^*^	87.3% in children [51]; 61.0% in pregnant women [8]	Poor pregnancy outcome, impaired growth (stunting), genetic disorders, decreased resistance to infectious diseases
Folate^*^	6.2% in children [52]; 12.0% in pregnant women [8]	Neural tube and other birth defects, megaloblastic anemia, heart disease, stroke, impaired cognitive function, depression
Vitamin A^*^	8.5% in children [52]; 7.0% in pregnant women [8]	Xerophthalmia (night blindness, Bitot's spot, corneal ulcer, keratomalacia, xerosis), increased risk of morbidity and mortality, increased risk of anemia
Vitamin D^*^	17.2% in children [52]; 14.0% in pregnant women [8]	Rickets, osteomalacia, osteoporosis, colorectal cancer
Vitamin E^*^	17.9% in children [52]; 25.0% in pregnant women [8]	Ataxia, peripheral neuropathy, muscle weakness, miscarriages, slow growth in children
Vitamin C^*^	Limited information	Scurvy (fatigue, hemorrhages, low resistance to infection, anemia)
Vitamin B1^*^	Limited information	Beriberi (cardiac and neurologic), Wernicke, and Korsakov syndromes (alcoholic confusion and paralysis)
Vitamin B2^*^	33.0% in pregnant women [8]	Nonspecific (fatigue, eye changes, dermatitis, brain dysfunction, impaired iron absorption)
Vitamin B3^*^	Limited information	Pellagra (dermatitis, diarrhea, dementia, death)
Vitamin B6^*^	43.1% in children [52]; 40.0% in pregnant women [8]	Dermatitis, neurological disorders, convulsions, anemia, elevated plasma homocysteine
Vitamin B12^*^	18.1% in children [52]; 28.0% in pregnant women [8]	Megaloblastic anemia (associated with *Helicobacter pylori* induced gastric atrophy)
Calcium^*^	Limited information	Decreased bone mineralization, rickets, osteoporosis
Selenium^*^	59.0% in children [52]	Cardiomyopathy and increased cancer and cardiovascular risk
Fluoride^*^	Limited information	Affects bone health including increased dental decay

^*^Lacking data from national survey.
